# Beyond Context‐Transfer Effects: Attenuated Familiarity During Virtual Reality‐Based Retrieval Across Different Encoding Modalities

**DOI:** 10.1111/ejn.70447

**Published:** 2026-03-06

**Authors:** Joanna Kisker, Marius Soethe, Merle Sagehorn, Thomas Gruber

**Affiliations:** ^1^ Experimental Psychology I Institute of Psychology, Osnabrück University Osnabrück Germany; ^2^ Institute for Medical Education University Hospital Essen, University of Duisburg‐Essen Essen Germany

**Keywords:** encoding specificity, ERP old/new effects, recognition memory, source memory, virtual reality

## Abstract

Whereas the circumstances under which episodic memory benefits from Virtual Reality (VR)‐based encoding remain elusive, preliminary findings suggest that the contributions of the underlying retrieval processes might depend on the encoding modality. Previous research indicates that engrams obtained from VR conditions elicit enhanced recollection alongside attenuated familiarity. However, it remains unclear whether this pattern depends on the congruence of the encoding and retrieval contexts. Consequently, this study examined potential context‐transfer effects on the electrophysiological correlates of familiarity and recollection after VR‐based and PC‐based encoding. A source memory paradigm was employed to test the retrieval of objects and their encoding context, i.e., item and source memory under VR conditions. The electrophysiological results indicated attenuated familiarity of PC‐based engrams reflected in the frontal old/new effect (FN400), yet the same held true for VR‐based engrams. Moreover, a strong old/new effect in the late positive component (LPC) linked to recollection was evident under both conditions. In contrast, the late posterior negativity (LPN), linked to the search for and reactivation of contextual details during retrieval, was observed under neither condition. In summary, the present results indicated comparable contributions of familiarity and recollection to retrieval, independent of the encoding modality, when retrieval occurred under VR conditions. While effects on engrams retrieved without their correct source might, to some degree, depend on context‐transfer effects, familiarity was attenuated across encoding modalities. Consequently, the present results demonstrate that disparities between VR‐ and PC‐engrams depend on the combination of encoding and retrieval modalities and extend beyond context‐transfer effects.

## Graphical abstract

The study examined potential context‐transfer effects on the relative contributions of familiarity and recollection to retrieval across virtual reality (VR) and desktop (PC) encoding conditions. Item and source memory were tested in a source memory paradigm employed in VR. The results suggest that retrieval of engrams without recalling their encoding context might be affected by context‐transfer, whereas the observed attenuation of familiarity goes beyond mere encoding specificity effects.

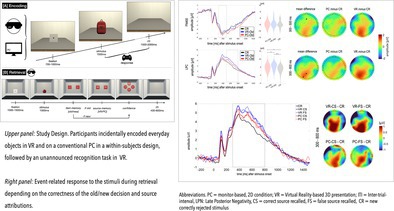

**Abbreviations**
CR: correct rejection
CS: correct source retrieved
ECG: electrocardiogram
EEG: electroencephalography
EOG: electrooculogram
FS: false source retrieved
HMD: head‐mounted display
ITI: inter‐trial‐interval
PC: personal computer
VR: virtual reality

## 1 Introduction

To date, the circumstances under which episodic memory benefits from virtual reality (VR)‐based encoding resist a straightforward identification (Smith 2019; Smith and Mulligan 2021). Superior retrieval performance was found for engrams formed under VR conditions (i.e., VR‐engrams) compared to those formed under conventional PC‐based conditions (i.e., PC‐engrams) concerning various encoded materials, for example, photorealistic scenes (Schöne et al. 2019, 2021), 2D stimuli presented within VR (Krokos et al. 2019), and descriptions and executions of actions (Harman et al. 2017). However, further results indicate equal memory performance under both conditions (Cadet and Chainay 2020; Ernstsen et al. 2019; Kisker et al. 2021b; Kisker, Soethe, et al. 2025). Re‐evaluating the trend proposing enhanced episodic memory retrieval after VR‐based encoding compared to PC‐based encoding (Serino and Repetto 2018; Smith 2019; Smith and Mulligan 2021), recent comparisons of VR‐ and PC‐engrams focus on the processes underlying retrieval rather than behavioral outcomes. In particular, behavioral outcomes result from the complex interplay of several cognitive processes, yet varying combinations thereof can lead to successful memory retrieval per se. Based on the dual‐process theory of recognition memory, it has been hypothesized that the extent to which the underlying processes, familiarity and recollection (Rugg and Curran 2007; Yonelinas 2002), are contributing to retrieval depends on the presentation medium used for encoding (Kisker et al. 2021a, 2021b; Schöne et al. 2019). Specifically, familiarity is usually considered an automatic retrieval process providing a general sense of recognition (Rugg and Curran 2007; Yonelinas 2002). In contrast, recollection is a more controlled process and involves the conscious retrieval of specific contextual details from encoding bound to the retrieved item (Diana et al. 2008; Eichenbaum et al. 2007; see also Rugg and Curran 2007; Yonelinas 2002). Although distinct, both processes are usually considered to complementarily contribute to recognition memory (Tulving 1985; Yonelinas 2002). However, initial findings suggest that the processes' involvement is not necessarily evenly matched but that the retrieval of PC‐engrams might more strongly rely on familiarity, whereas that of VR‐engrams might more strongly rely on recollection (Kisker et al. 2021a, 2021b; Schöne et al. 2019).

The association of VR‐engrams with recollection has been proposed to result from VR's immersive features, which offer a sensory‐rich context, including spatial and temporal features considered defining episodic memory (Parsons 2015; Serino and Repetto 2018; Smith 2019). Moreover, the use of VR head‐mounted displays (HMDs) provides sensory cues in a natural and integrated manner. For example, vestibular (e.g., balance, acceleration) and proprioceptive input (e.g., body position) are congruently mimicked by head tracking, whereas desktop setups are restricted in providing these embodied cues (Costes and Lécuyer 2022; Santos et al. 2008). Although the latter cues are most beneficial for spatial memory tasks, HMDs are proposed to support, e.g., natural spatial awareness, presence, and memory recall (Costes and Lécuyer 2022; Krokos et al. 2019; Santos et al. 2008).

However, the aforementioned studies comparing memory retrieval across encoding modalities differentiated the underlying processes using old/new or remember/know paradigms related to item memory, i.e., remembering a specific item previously encoded (see, e.g., Gold et al. 2006; Rugg et al. 2012). In these paradigms, participants are asked to differentiate between items encoded in a preceding study phase and new, unknown items (old/new task) or to discriminate further whether the item was recognized by familiarity or recollected by recalling additional contextual information (remember/know task; e.g., Tulving 1985). However, the dissociation of retrieval processes in the latter paradigm depends on participants' subjective understanding and awareness of these processes (Yonelinas 2002), whereas the accuracy of both is seldom controlled for (Migo et al. 2012). Consequently, the conclusions drawn about distinctions in the retrieval processes underlying VR‐ and PC‐engrams remain indirect and limited by the subjective categorization of items as familiar or recollected.

Overcoming this limitation, item memory tasks have been complemented with source identification tasks. These tasks objectively discriminate recollection from item familiarity by testing contextual details linked to the encoding episode, i.e., source memory (Jacoby 1991; Kwon et al. 2023; Wynn et al. 2024). Associative information considered to contribute to source memory includes specifying elements of the retrieval context, like spatiotemporal information (Bröder and Meiser 2007) or the encoding modality (Johnson et al. 1993). Recalling a stimulus bound to additional contextual information from the encoding episode, i.e., its source, is considered to rely on recollection. Albeit familiarity can contribute to the retrieval of context information under certain conditions (Addante et al. 2024; Eichenbaum et al. 2007; Yonelinas et al. 2010), remembering a stimulus by familiarity does usually not include the recall of source‐specifying information (Kwon et al. 2023; Migo et al. 2012). Specifically, recent advancements distinguish between familiarity with an item, in which case no associated source information is retrieved, and familiarity with context information, which is linked to uncertainty about the retrieval of the respective item (Addante et al. 2024).

Consequently, the question arose whether the presentation medium used for encoding, i.e., VR or 2D desktops, is bound to the stimulus as relevant source information, fostering recollection‐based memory, and whether differences in source retrieval might contribute to the differences reported in recognition memory between VR‐ and PC‐based engrams. Against this background, the current study maintains a focus on item familiarity rather than context familiarity, and on whether the presentation medium is retrieved as a source of information linked to the stimulus. In a previous study (Kisker, Soethe, et al. 2025), we examined whether and to what extent the encoding modality modulates retrieval processes when only dimensionality is varied, specifically contrasting 3D stimulus presentation in VR with 2D stimulus presentation on a desktop. To this end, participants encoded everyday objects three‐dimensionally in VR or two‐dimensionally on a monitor (PC condition), followed by a recognition memory task including old/new judgements to examine item memory and source identifications (VR/PC) to examine source memory. To foster the objectification of previous behavioral results, we focused on analyzing the electrophysiological correlates of recognition memory. Item familiarity is associated with a frontal old/new effect in the event‐related potential (ERP), i.e., it is less negative‐going for old than for new stimuli. This difference effect occurs ~300–500 ms after stimulus onset and is also known as the FN400 (Kwon et al. 2023; Mecklinger 2000; Rugg and Curran 2007). In contrast, recollection is associated with a more positive going response to old than to new stimuli, as measured at parietal sensors. This old/new effect occurs ~500–800 ms after stimulus onset and is labeled the late positive component (LPC; Johansson and Mecklinger 2003; Kwon et al. 2023; Rugg and Curran 2007). These distinct neural substrates are most often tied to item memory tasks and were complemented by the ERP deflection associated with source memory: The reactivation of source‐specific attributes, i.e., context information, is associated with the late posterior negativity (LPN) (Addante et al. 2024; Cycowicz et al. 2001; Herron 2007; Johansson and Mecklinger 2003; Mecklinger et al. 2016). Depending on its specific timing, the LPN reflects slightly different processes. The early LPN (approx. 700–1200 ms after stimulus onset) is related to the active search and reconstruction of episodic details, while the later LPN (approx. 1200–1800 ms) mirrors the evaluation and maintenance of the retrieved information. Thus, while the early LPN is more closely related to the immediate retrieval process, its later stages are associated with source monitoring and post‐retrieval processing (Herron 2007; Johansson and Mecklinger 2003; Mecklinger et al. 2007, 2016). Albeit the divergent definitions of the LPN vary in whether they ultimately refer to the search, the reconstruction, or the amount of details available from the encoding context, they overlap in linking it to the retrieval of contextual information of the encoding episode (see e.g., Addante et al. 2024; Johansson and Mecklinger 2003; Mecklinger et al. 2007, 2016). Although a matter of debate, it is not necessarily sensitive to the correctness of source retrieval (Johansson and Mecklinger 2003).

In summary, the results of the preceding study (Kisker, Soethe, et al. 2025) indicated that encoding modality had a limited impact on item memory, with no significant differences observed between encoding modalities in recognition performance or the related ERP markers, i.e., the FN400 and the LPC. However, the LPN responses differentiated between encoding conditions depending on the accuracy of the source identifications. In detail, stimuli correctly recognized as old and recalled with their correct source are considered to be based more strongly on recollection, i.e., including but going beyond item familiarity. In contrast, responses to stimuli correctly recognized as old but without their correct source are considered to predominantly reflect item familiarity (Kwon et al. 2023; Migo et al. 2012). Consequently, an electrophysiological estimate of recollection was obtained by subtracting the ERP responses to stimuli with incorrect source attributions from those with correct source attributions, and an electrophysiological estimate of item familiarity was obtained by subtracting the ERP responses to correctly rejected stimuli from the responses to stimuli without correct source attributions. These estimates based on the ERPs, indicated enhanced contributions of recollection to the retrieval of VR‐engrams alongside attenuated contributions of familiarity, particularly when source retrieval failed. Hence, although encoding modality served as a relevant source for recollection to some extent, i.e., could be retrieved bound to the item, familiarity as reflected in the ERP seemed more sensitive to contextual discrepancies in stimulus dimensionality (Kisker, Soethe, et al. 2025).

However, this study mandatorily implemented the retrieval task on a conventional screen for both kinds of engrams. This rationale allowed attributing the observed results to the experimental manipulations during encoding, while accounting for similar sensory input across all conditions during retrieval. Since successful retrieval depends to some degree on the congruence of the encoding context and the retrieval context, the results might be affected by context‐transfer effects (Godden and Baddeley 1975; Johnsdorf et al. 2025; Morris et al. 1977; Shin et al. 2021; Tulving and Thomson 1973). In particular, the results may reflect a mismatch between the 3D encoding of VR‐engrams and 2D retrieval cues, thereby reducing access to contextual information. However, a previous study indicated that although the congruence of encoding and retrieval contexts led to equal memory performance in VR and desktop experiences, VR experiences retrieved on a desktop yielded equally high memory performance, indicating sufficient processing to mitigate potentially negative effects of contextual mismatch on behavioral outcomes (Johnsdorf et al. 2025).

To synthesize these findings, the present study aimed to control for potential context‐transfer effects on the electrophysiological correlates of item familiarity and recollection. To this end, we replicated the preceding study's paradigm but crucially modified the recognition memory task by implementing it as a VR condition for the retrieval of both VR‐ and PC‐engrams. This variation was explicitly designed to investigate the role of the contextual match between encoding and retrieval across modalities: If the findings of the preceding study (Kisker, Soethe, et al. 2025) were predominantly based on context‐transfer effects, we expected a reversal of the respective effects in the present study. In detail, we expected that PC‐based engrams might afford stronger recollection and attenuated familiarity when recalled in VR. In contrast, the retrieval of VR‐based engrams might not yield different levels of recollection and familiarity. Both of the latter should be reflected in a modality‐dependent modulation of the LPN. In contrast, we expect that PC‐engrams retrieved without their source will stand out under VR‐based retrieval, i.e., to yield no significant difference in the LPN compared with correctly identified new items. This outcome would support the conclusion that contextual factors may drive the reported modality effects and should thus be accounted for in future studies. Regarding item memory, we hypothesize that the FN400 and LPC will indicate similar engagement of familiarity and recollection, respectively, across encoding modalities. Based on the idea that potential facilitating effects of VR‐based encoding on memory performance were diminished by a mismatch between encoding and retrieval contexts in the preceding study (Johnsdorf et al. 2025; Kisker, Soethe, et al. 2025), we expected better memory performance for VR‐engrams compared to PC‐engrams when retrieved under VR conditions, reflected in higher d‐prime (*d'*) and accuracy values.

## 2 Methods

### 2.1 Sample Size and Participants

The study's procedures were approved by the local ethics committee of Osnabrück University (Ethik‐36/2024). Participants provided informed written consent and were unaware of the specific hypotheses under investigation. Participation was compensated with either partial course credit or monetary compensation. A power analysis was performed using G* Power (Faul et al. 2007). Based on the preceding study, the power calculation was based on a 1 × 5 repeated measures ANOVA (rmANOVA) design, and the effect size was estimated at *f = 0*.37, i.e., calculated from the rmANOVA applied for the LPN with an effect size of *η* ^*2*^ = 0.12 in the previous study (Kisker, Soethe, et al. 2025). The power analysis indicated a required sample size of 15 participants. To account for possible technical challenges and ensure consistency with existing VR literature, a target sample size of 30 participants was ultimately adopted. Consequently, 30 participants were recruited from Osnabrück University and screened for psychological or neurological conditions. One participant was excluded because they did not meet the inclusion criteria, and the data of another person were collected only for piloting. The final sample for analyses thus comprised 28 data sets (*M*_age_ = 21.39; SD_age_ = 3.47; 24 right‐handed; sex: 22 female, six male; all cisgender). 19 participants had prior experience with VR HMDs, but none used them regularly. 13 participants used computers regularly for entertainment, i.e., gaming. While the behavioral data of all 28 participants could be included in analyses, three datasets had to be excluded from electrophysiological analyses due to severe artifacts (see Section 2.5 *Electrophysiological recordings and preprocessing*).

### 2.2 Stimulus Material

**FIGURE 1:**
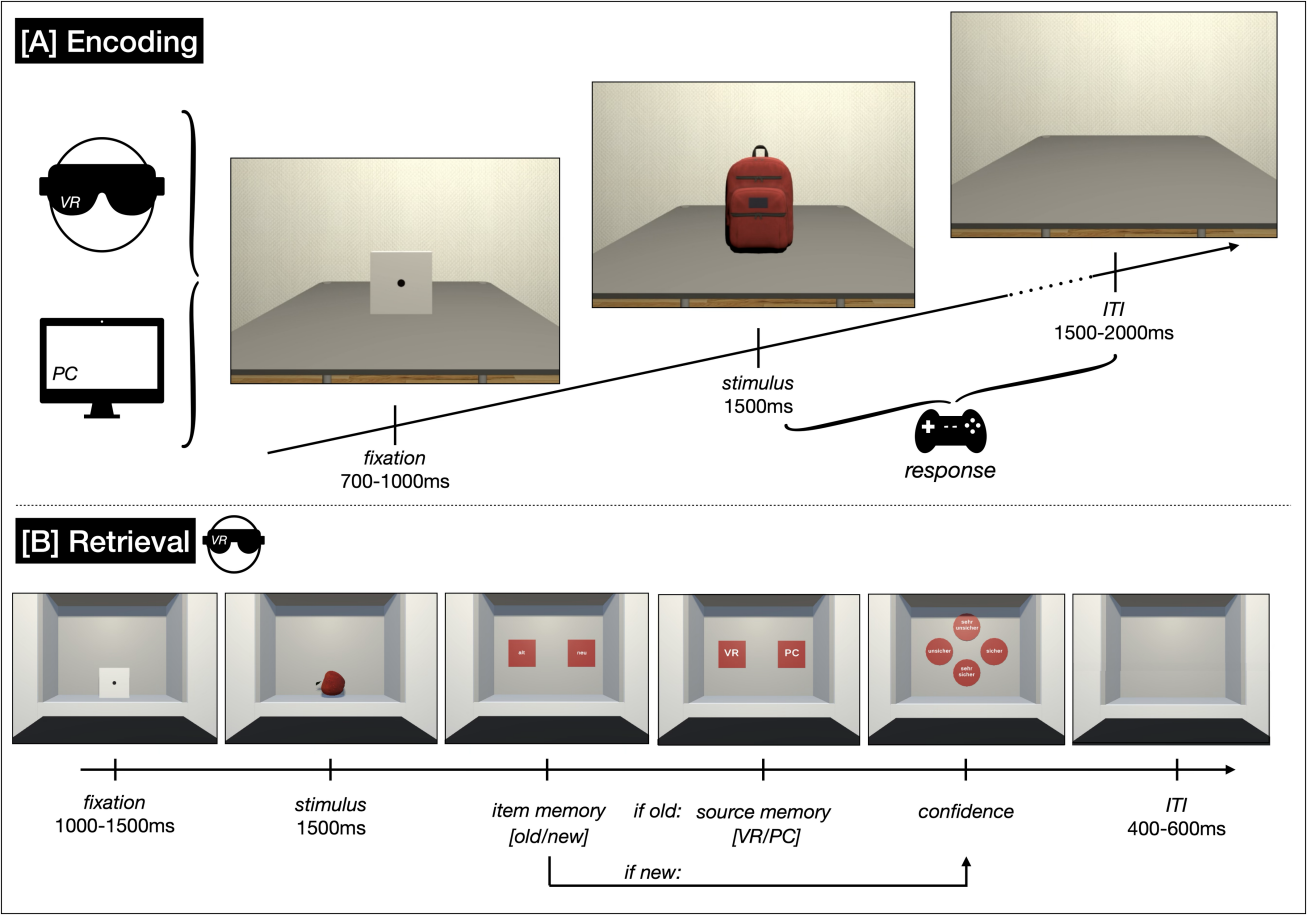
Schematic representation of the experimental procedure. *Note:* [A] Participants encoded 100 stimuli each in VR and on a PC in a within‐subjects design. The encoding phase took approximately 25 min in total, including a 2‐min break after 50 trials per condition. [B] The retrieval task took approximately 50 min, including two breaks. ITI = Inter‐trial‐interval.

The stimuli were 305 3D objects obtained from three existing databases (Downs et al. 2022; Peeters 2018; Tromp et al. 2020) because none of the databases alone contained enough (semantically) different objects for the study. Five of these objects were used for training trials (see Section 2.3). The 300 remaining objects were split into two categories based on size: half represented items that would fit in a shoebox, considering their real‐life size, and half represented items too large for a shoebox (see behavioral task and Figure 1).

### 2.3 Procedure

The study follows up on a preceding study from our working unit and employed the same encoding phase, while the retrieval phase was varied by employing a VR condition instead of a (2D) desktop condition (Kisker, Soethe, et al. 2025). The experiment adhered to established source memory designs, starting with an incidental encoding phase followed by an unannounced recognition memory test in a within‐subjects design (see e.g., Gruber et al. 2008). In the encoding task, participants were asked to estimate whether each object would fit into a shoebox based on its real‐life size. The encoding phase comprised two conditions, a VR condition and a conventional desktop condition (PC). Both conditions were presented in a randomized order and were developed using Unity 5 (version 2023.2.1f1; Unity Technologies, San Francisco, USA). The field of view of the virtual camera was set to the default of 60° in both conditions.

The VR condition was presented using an HTC Vive Pro 2 HMD, providing a per‐eye resolution of 2448 × 2448 pixels and a frame rate of 90 Hz. Participants were positioned seated at a grey virtual table, 90 cm from the stimulus presentation location. The PC condition was presented using a 55″ display positioned 70 cm from the participant, offering a resolution of 1920 × 1080 pixels and a frame rate of 60 Hz. The same virtual environment, including the table, was rendered in 2D for this condition. The visual angle was aligned across conditions using the size of the fixation cube as a reference (see Figure 1). The cube was displayed on the desktop in the PC condition measuring 15 cm × 15 cm, and in the VR condition measuring 19 cm × 19 cm. For each condition, the distance between the participants and the cube was adjusted to achieve a viewing angle of 12°.

Each condition comprised five training trials and 100 experimental trials. For each participant, the 300 stimuli chosen from the databases were randomly allocated to three subsets: 100 VR encoding trials, 100 PC encoding trials, and 100 distractor items for the subsequent recognition memory task. Within each subset, 50 objects were larger and 50 objects were smaller than the dimensions of a standard shoebox, based on real‐life size estimations.

Before the experimental trials, participants were verbally instructed and allowed to address any task‐related uncertainties. To mitigate potential fatigue and muscular strain, a two‐minute break was automatically scheduled after every 50 experimental trials. Each trial started with a fixation (700–1000 ms), followed by stimulus presentation (1500 ms) and an inter‐trial interval (ITI, 1500–2000 ms; see Figure 1). Participants were instructed to respond as quickly as possible by pressing the shoulder buttons on a standard gamepad to indicate whether each object's real‐world size was larger or smaller than a conventional shoebox. Button assignments for “larger” and “smaller” responses were counterbalanced across participants. Trials were paused until a response was registered, even if the stimulus had previously disappeared. The training initiation, the start of the experimental trial, and the break offset were signaled visually by a green cube displaying a start symbol. Training completion, break onset, and the experiment's end were indicated by a red cube depicting a pause symbol. Each condition required approximately 12 min to complete, including the scheduled break.

After participants completed both encoding conditions, they were relocated to the electroencephalogram (EEG) laboratory for preparation of a 32‐channel EEG. To minimize the potential confounding effects of rehearsal on the subsequent recognition task, participants were asked to complete a Sudoku puzzle on a tablet during EEG setup. Participants continued working on the Sudoku until either successful completion or a 10‐min time limit was reached, with the earlier event serving as the cut‐off criterion.

EEG was recorded during the recognition memory task, during which participants were seated in an electrically shielded cabin (Faraday cage). All participants completed this task in VR. The task was programmed using Unity 5 (version 2023.2.1f1; Unity Technologies, San Francisco, USA). Participants were seated in front of a wall with a shelf‐like recess, where the objects were presented in 3D (see Figure 1, Retrieval). As in the previous study, the environment did not visually replicate the encoding environment. The instructions were given verbally before the VR HMD was put on. Five training trials were conducted to clarify remaining uncertainties before the experimental trials began. The unannounced recognition test comprised the total set of 300 3D objects. Objects presented during encoding in VR are subsequently denoted as “VR”, objects presented during encoding on the PC screen as “PC”, and objects that were only presented during the retrieval task, and are thus unknown, are denoted as “new”. The viewing angle was calculated for each object and participant by tracking the distance between the headset position and the stimulus. The viewing angle was calculated for each stimulus at the time of its onset and averaged across trials and participants, resulting in an average viewing angle of 10.12° (±4.08°).

Each trial of the recognition task began with a 1000–1500 ms fixation period, followed by 1500 ms of stimulus presentation. Upon stimulus offset, a series of rating scales was presented sequentially (see Figure 1, Retrieval). Participants initially indicated stimulus recognition via an old/new judgment (item memory). For stimuli identified as “old”, a subsequent source identification task assessed whether participants remembered having encoded the item in the VR or PC condition (source memory). Stimuli categorized as “new” bypassed the source identification task. Finally, participants rated their confidence in their response on a 4‐point Likert scale (1 = *very unsure*, 2 = *unsure*, 3 = *sure*, 4 = *very sure*). This series of ratings was followed by a 600 ms ITI (blank screen). The entire recognition task required approximately 50 min. To mitigate fatigue, participants were offered a break after every 100 trials, and took an average of 42 and 60 s for breaks following 100 and 200 trials, respectively (average total break duration: 101 s). Upon completion of all trials, the VR and EEG equipment were removed, and participants were debriefed.

### 2.4 Behavioral Data

Response time and accuracy during the encoding phase were calculated separately for the VR and PC encoding conditions. Response time was measured as the time, in milliseconds, between the onset of the stimulus and the participants' response. Accuracy was quantified as the proportion of correct responses relative to all responses in the encoding task.

To determine the effect of encoding modality on memory, we quantified item memory performance by calculating the proportion of correctly identified old items for both VR‐ and PC‐encoded stimuli. Correspondingly, source memory performance was determined as the ratio of correct source attributions to the total number of ratings per modality, alongside an evaluation of the confidence associated with those attributions. *d'* was calculated as a measure of sensitivity—that is, the participant's ability to distinguish between previously seen items and novel distractors (*d'* = *z (hits)—z (false alarms)*; Swets et al. 1961). Moreover, criterion *C* was calculated (*C = −*0.5 * *(z (hits) + z (false positive responses))*, Hautus et al. 2021; Macmillan 2002) per encoding modality and task to control for potential response biases. This measure indicates whether participants tend to give a particular answer if uncertain. Negative values of *C* indicate liberal biases, with a tendency toward higher hit rates and false alarm rates. Positive values indicate conservative biases, with a tendency toward more correct rejections (CRs) and fewer misses (Macmillan 2002).

Further analyses examined the confidence ratings associated with judgments made during the source identification task, contrasting conditions representing correct source attribution of PC‐engrams (PC‐CS), false source attribution of PC‐engrams (PC‐FS), correct source attribution of VR‐engrams (VR‐CS), false source attribution of VR‐engrams (VR‐FS), and CRs.

### 2.5 Electrophysiological Recordings and Preprocessing

EEG data were acquired using a 32‐active‐electrode system from Biosemi (Amsterdam, Netherlands) during the recognition task of the experiment. Electrode placement followed the standardized international 10–20 system, with additional Common Mode Sense (CMS) and Driven Right Leg (DRL) electrodes employed as online reference and ground, respectively. Before the recognition task, the live EEG signal was inspected and corrected for line noise and slow drift artifacts to ensure an optimal signal‐to‐noise ratio. Data were recorded at a sampling rate of 512 Hz, and an online bandpass filter (0.016–100 Hz) was applied during recording. Subsequent preprocessing of the EEG data was conducted in MATLAB (version R2024a, MathWorks Inc.) using the EEGLAB toolbox (version 2024, Delorme and Makeig 2004).

The raw continuous data were filtered using the FIR bandpass filter implemented via the respective EEGLAB function, with parameters set to 0.25 Hz high‐pass and 30 Hz low‐pass. The data at each electrode were independently detrended and segmented into epochs from 500 ms before stimulus onset to 1500 ms after stimulus onset. Baseline correction was subsequently applied, utilizing the −500 ms to stimulus onset interval. A single flatlined channel was identified for one participant and interpolated. Blink‐contaminated trials were quantified and removed (*M* = 44.14, SD = 20.77) using *statistical control of artifacts in dense array studies* (*SCADS*; Junghöfer et al. 2000). *Independent component analysis* (*ICA*) was performed, with artifactual components identified and removed employing the ICLabel algorithm (Pion‐Tonachini et al. 2019) based on thresholds for eye movements (> 0.7), muscle activity (> 0.9), and electrocardiographic (ECG) artifacts (> 0.9). After removal of the respective components (*M* = 1.61, *SD* = 1.37), the data were re‐referenced to the average reference. Post‐preprocessing signal quality was assessed by visualizing the root‐mean‐square (RMS) amplitude across all electrodes for each participant. If the P1 component was not clearly identifiable in the RMS, the respective dataset was excluded from analyses of the EEG data. Based on this assessment, data from three participants were excluded due to unacceptable quality, resulting in a final participant count of *n* = 25 for subsequent ERP analyses. With respect to the old/new task, the average number of artifact‐free trials was 63.75 (*SD* = 10.42) for PC‐engrams (PC‐Old), 65.57 (*SD* = 8.26) for VR‐engrams, and 73.79 trials (*SD* = 10.75) for correctly rejected new items (CR). Regarding the source identification task, 39.75 trials (*SD* = 9.55) were available for VR‐engrams with correct, and 25.82 trials (*SD* = 7.25) for false source retrieval. 37.86 trials (*SD* = 10.70) were available for PC‐engrams with correct, 25.89 trials (*SD* = 8.36) for false source retrieval, and 73.79 trials (*SD* = 10.75) regarding CRs of new objects. The trial ranges for each condition are provided in Table S1.

The time windows and regions of interest were derived from the most recent review (Kwon et al. 2023) and the preceding study (Kisker, Soethe, et al. 2025), indicating an old/new effect in the time window from 300 to 500 ms after stimulus onset at frontal sensors (FN400) and a partial old/new effect from 500 to 800 ms after stimulus onset at parietal sensors (LPC; Kwon et al. 2023; see also Mecklinger 2000; Rugg and Curran 2007). The electrodes for analyses were counterchecked against the mean difference between conditions for the respective time window and the maximum regional means. Accordingly, in line with previous literature (e.g., Kwon et al. 2023; Mecklinger 2000; Rugg and Curran 2007), the electrode Fz was chosen for analyses regarding the early time window, and amplitudes were averaged across Pz and PO3 for analyses of the later time window. The time window and region of interest were also derived from previous literature and the preceding study for the LPN, suggesting a source memory effect in the LPN linked to the immediate retrieval process from 700 to 1200 ms after stimulus onset and maximal around Pz and POz (Johansson and Mecklinger 2003; Mecklinger et al. 2007, 2016). Surprisingly, no negative maximum was observed across conditions. Consequently, the electrodes Pz, CP1, CP2, PO3, and PO4 were chosen for analysis based on the positive maximum instead. However, these electrodes still align with the electrodes usually included for analyses of the LPN. A visual inspection indicated a potential differentiation of the conditions in an earlier time window than hypothesized. Thus, an exploratory analysis of the time window from 300 to 800 ms after stimulus onset, based on the same regional mean, was included. Since the original power analysis was based on the assumption that the LPN would be analyzed using a 1 × 5 rmANOVA, the power analysis was rechecked for a 2 × 5 rmANOVA. This countercheck indicated a required sample size of *n* = 20. Hence, the inclusion of the additional time window into analyses is not limited by our sample size.

### 2.6 Statistical Analyses

As the general approach, inferential statistics were carried out and complemented by the effect sizes partial eta squared (*η* ^2^) and Cohen's *d*. Additionally, the Bayes Factor (*BF*_10_) was calculated for each rmANOVA and post hoc *t*‐test to provide more robust conclusions about differences or similarities between conditions. Standard deviation and effect size are reported in the main text, whereas a comprehensive report of descriptive statistics on the post hoc *t*‐tests can be found in the supplementary material (Tables S2 and S3).

JASP (JASP Team 2024; Kelter 2020) was used to calculate the *BF*_10_. A *BF*_10_ > 1 favors the H_1_, while a *BF*_10_ < 1 favors the H_0_. The following thresholds were applied as recommended for JASP (Kelter 2020): *BF*_10_ < 1—anecdotal evidence for H0, *BF*_10_ < 0.33—moderate evidence for H_0_, *BF*_10_ < 0.10—strong evidence for H_0_, *BF*_10_ < 0.03—very strong evidence for H0; *BF*_10_ > 1—anecdotal evidence for H_1_, *BF*_10_ > 3—moderate evidence for H_1_, *BF*_10_ > 10—strong evidence for H_1_, *BF*_10_ > 100—very strong evidence for H_1_.

#### 2.6.1 Behavioral Data

Response times during encoding were compared using a 2 × 2 rmANOVA with the factors *Encoding Modality* (VR, PC) and *Correctness* (correct answer, false answer). Post hoc tests were carried out as two‐tailed paired‐sample *t*‐tests. Since only one *t*‐test was carried out based on this rmANOVA, no correction for multiple testing was applied. The number of correct responses during encoding was compared between VR‐based and PC‐based encoding using two‐tailed paired‐sample *t*‐tests. The parameters operationalizing memory performance (see Section 2.4) were compared between VR and PC using one‐tailed paired‐sample *t*‐tests. The significance threshold was Bonferroni‐corrected to 0.017. The participants' confidence in their ratings was compared using a 1 × 5 rmANOVA with the within‐subjects factor *Condition* (VR‐CS, VR‐FS, PC‐CS, PC‐FS, and CR) and followed by two‐tailed paired‐samples *t*‐tests. To facilitate direct comparison with the ERP results, confidence levels across all kinds of engram were compared with CRs, as in the established procedure for the electrophysiological data. Additionally, comparisons between conditions were made to examine which engram was retrieved with the highest confidence, depending on the encoding modality and source identification. The significance level was Bonferroni‐corrected to 0.006. Criterion *C* was tested against zero per encoding modality and task, using two‐tailed one‐sample *t*‐tests, and compared between encoding modalities per task using paired‐sample *t*‐tests.

#### 2.6.2 ERP

With respect to the item memory task, the trials were segregated into separate files for correctly identified old PC objects (PC‐Old), correctly identified old VR objects (VR‐Old), and correctly rejected new objects (CR). To analyze the early and late old/new effects, i.e., the FN400 and the parietal LPC, a 1 × 3 rmANOVA with the within‐subjects factor *Condition* (VR‐Old, PC‐Old, and CR) was used. If the rmANOVA indicated a significant effect of *Condition*, post hoc *t*‐tests for paired samples were applied for comparisons between conditions. According to the hypotheses, they were carried out one‐tailed. The significance threshold for the post hoc *t*‐test was corrected to 0.029 using the false discovery rate (FDR) according to the Benjamini‐Hochberg method (Benjamini and Hochberg 1995).

Regarding the source identification task, the trials were segregated into separate files for VR objects with correct (VR‐CS) or false source retrieval (VR‐FS), PC objects with correct (PC‐CS) or false source retrieval (PC‐FS), and CRs of new objects (CR). Consequently, to analyze the LPN effect, a 2 × 5 rmANOVA with the within‐subject factors *Time Window* (300–800 ms, 700–1200 ms) and *Condition* (VR‐CS, VR‐FS, PC‐CS, PC‐FS, CR) was conducted. Whenever necessary, the Greenhouse–Geisser correction was applied. In the case of a significant interaction between both factors, a 1 × 5 rmANOVA including only the factor *Condition* was carried out for each time window. Only when the follow‐up rmANOVA indicated a significant effect of *Condition* within the respective time window, post hoc *t*‐tests for paired samples were applied to prevent an accumulation of post hoc tests. Each old condition (VR‐CS, VR‐FS, PC‐FS, and PC‐CS) was compared individually with CR (see Gruber et al. 2008). In case the 1 × 5 rmANOVA for the time window of the LPN (700–1200 ms) yields significant results, electrophysiological estimates of recollection and familiarity by means of the ERPs were planned to be calculated based on the most recent review (Kwon et al. 2023). As outlined in the introduction, subtracting the ERP responses to stimuli with incorrect source attributions (FS) from those with correct source attributions (CS) yields an estimate of recollection (VR‐recollection, PC‐recollection). An estimate of item familiarity (VR‐familiarity, PC‐familiarity) is obtained by subtracting the ERP responses to correctly rejected stimuli (CR) from the response to stimuli without correct source attributions (FS). Please note that we originally planned to analyze these estimates based on the ERPs, but ultimately did not include them in the analysis due to the outcome of the underlying 1 × 5 rmANOVA (see Section 3.3.2, *source identification task*). The significance threshold for post hoc *t*‐tests was FDR‐corrected to 0.019. Based on the hypotheses, one‐tailed paired‐samples *t*‐tests were conducted.

## 3 Results

### 3.1 Encoding Phase

A significant effect of *Correctness* (*F*(1, 27) = 29.88, *p* < 0.001, *η* ^2^ = 0.53; *BF*_10_ = 2351.04) was found for the response times to the encoding task, while no effect of *Encoding Modality* (*F*(1, 27) = 0.43, *p* = 0.517, *η* ^2^ = 0.02; *BF*_10_ = 0.30) and no interaction between both factors (*F*(1, 27) = 0.026, *p* = 0.874, *η* ^2^ < 0.01; *BF*_10_ = 216.80) was evident. Correct answers were given approximately 255 ms faster than false answers (*t*(27) = −5.47, *p* < 0.001, *d* = 1.03; *M*_correct_ = 1046 ms, SD_correct_ = 193 ms, *M*_false_ = 1301 ms, SD_false_ = 341 ms, *BF*_10_ = 2414.88). The number of correct answers did not differ between VR‐based and PC‐based encoding (*t*(27) = −1.39, *p* = 0.176, *d* = 0.26; *M*_VR_ = 81.29, SD_VR_ = 11.36, *M*_PC_ = 83.86, SD_PC_ = 9.49, *BF*_10_ = 0.48).

### 3.2 Behavioral Memory Performance

Participants correctly recognized around 75% of previously encoded objects regardless of the encoding modality (*t*(27) = −1.71, *p* = 0.040, *d* = 0.32; *M*_VR_ = 0.74, SD_VR_ = 0.10, *M*_PC_ = 0.77, SD_PC_ = 0.08, *BF*_10_ = 0.73), and 86% of the new items were correctly rejected. Albeit *d'* indicated a trend towards better retrieval performance regarding VR‐engrams compared to PC‐engrams, it did not reach significance after Bonferroni correction (*t*(27) = 1.79, *p* = 0.042, *d* = 0.34; *M*_VR_ = 1.13, SD_VR_ = 0.40, *M*_PC_ = 1.10, SD_PC_ = 0.40, *BF*_10_ = 0.819), and an equal level of correct source ratings was achieved for both kinds of engrams (*t*(27) = 0.35, *p* = 0.733, *d* = 0.70; *M*_VR_ = 60.38%, SD_VR_ = 0.13%, *M*_PC_ = 59.07%, SD_VR_ = 0.11%, *BF*_10_ = 0.21).

**FIGURE 2:**
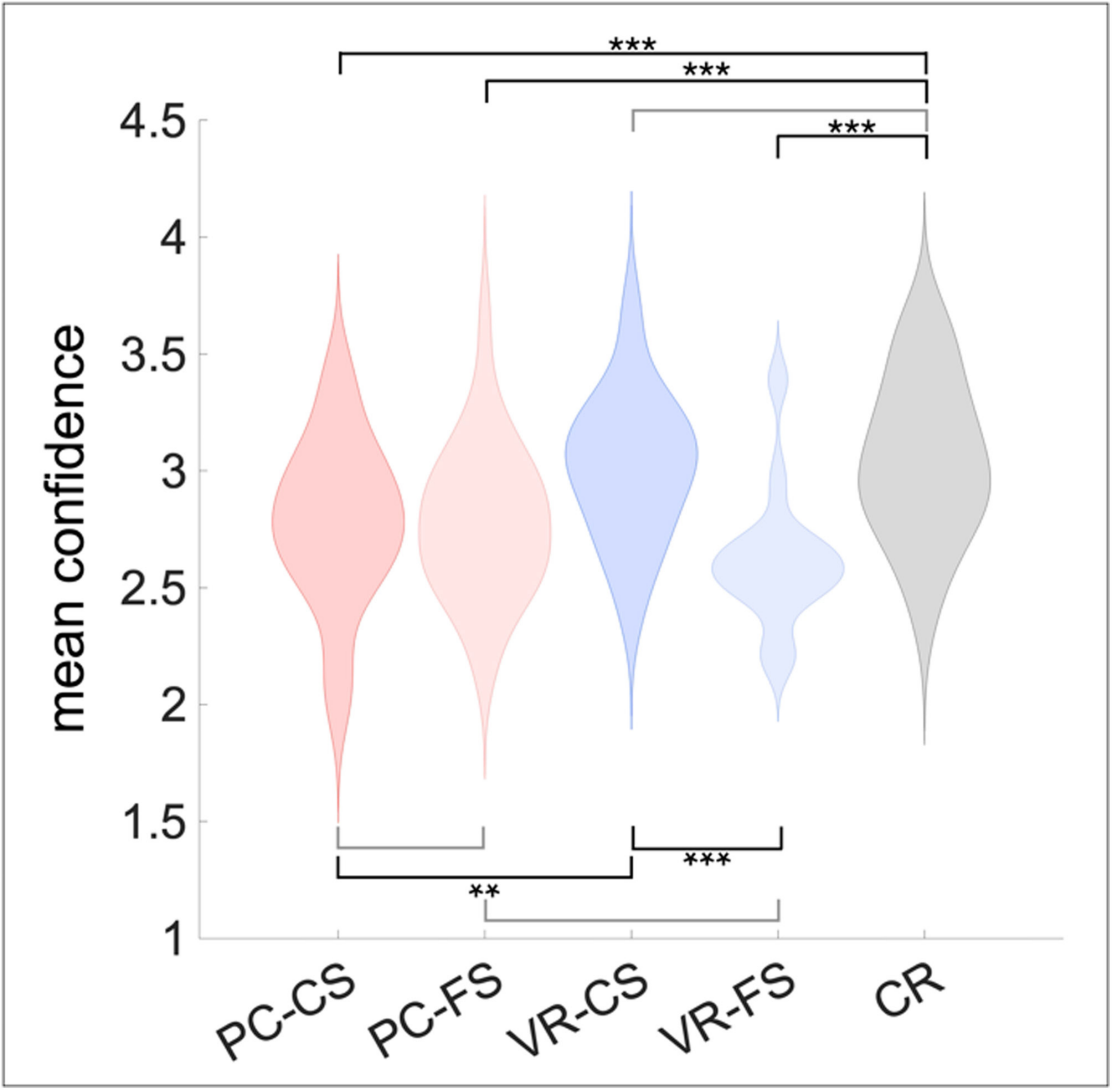
Participants' mean confidence in their ratings across source identification task conditions. Note. The confidence scale was a 4‐point Likert scale ranging from *very unsure* [1] to *very sure* [4]. Comparisons significant after Bonferroni‐correction (*p* < 0.006) are marked with **p < 0.006, ***p < 0.001. Light‐grey connecting lines indicate comparisons that did not reach significance after correction for multiple comparisons.

However, participants' confidence in their ratings differed across conditions (*F*(2.8, 76.14) = 17.91, *p* < 0.001, *η* ^2^ = 0.40; *BF*_10_ > 9999). Participants were most confident regarding correctly rejected items and VR‐items recalled with correct source, followed by both kinds of PC‐engrams, and lastly, by VR‐items recalled with incorrect source. Please refer to Table 1 and Figure 2 for the full report of the statistics.

**TABLE 1:** Test statistics for participants' confidence ratings.

Two‐tailed paired‐samples *t*‐tests				
Comparison	*t*(27)	*p*	*d*	*BF*_10_
CR vs. VR‐CS	0.63	0.532	0.116	0.241
CR vs. VR‐FS	7.65	< 0.001	1.405	441349.95
CR vs. PC‐CS	4.19	< 0.001	0.769	107.75
CR vs. PC‐FS	4.42	< 0.001	0.812	190.152
VR‐CS vs. VR‐FS	6.74	< 0.001	1.247	52885.91
PC‐CS vs. PC‐FS	−0.02	0.987	0.003	0.20
VR‐CS vs. PC‐CS	3.14	0.004	0.577	9.93
VR‐FS vs. PC‐FS	−2.84	0.009	0.521	5.28

*Note:* The significance level was Bonferroni‐corrected to 0.006.

Criterion *C* indicated no significant response bias regarding the old/new task for either encoding modality (*t*_*VR*_ (27) = 0.001, *p* = 0.999, *d* = 0.00; *BF*_10_ = 0.20, *M*_*VR*_ < 0.001, SD_VR_ = 0.58; *t*_*PC*_ (27) = 0.003, *p* = 0.998, *d* = 0.00; *BF*_10_ = 0.20, *M*_*PC*_ < 0.001, SD_PC_ = 0.64), as well as regarding the source task (*t*_*VRSource*_ (27) = −0.001, *p* = 0.999, *d* = 0.00; *BF*_10_ = 0.20, *M*_*VRSource*_ < −0.001, SD_VRSource_ = 0.32; *t*_*PCSource*_ (27) = −0.003, *p* = 0.998, *d* = 0.00; *BF*_10_ = 0.20; *M*_*PCSource*_ < 0.001, SD_PCSource_ = 0.51). Hence, no indication that participants tended to give a particular response when uncertain was found regarding either task. Furthermore, the comparison of both encoding conditions revealed no significant differences (old/new task: *t*(27) = −0.003, *p* = 0.998, *d* = 0.00; *BF*_10_ = 0.20; source task: *t*(27) = 0.003, *p* = 0.998, *d* = 0.00; *BF*_10_ = 0.20), indicating that neither encoding condition was significantly more biased than the other.

### 3.3 Event‐Related Potentials

#### 3.3.1 Item Memory Task

**FIGURE 3:**
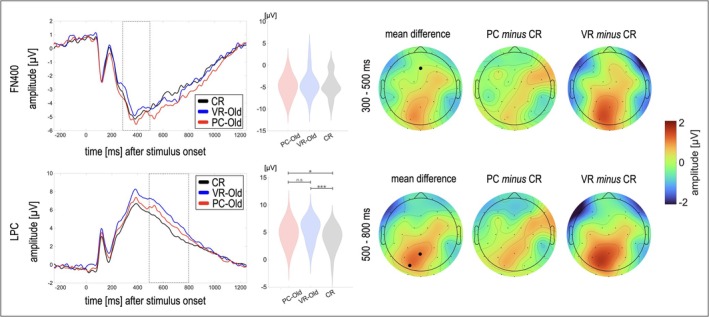
Amplitude distribution and line plots of the old/new effects. *Note.* The black dots in the topographies indicate the electrodes used for statistical comparisons of the conditions (FN400: Fz; LPC: Pz, PO3), and the grey dotted lines indicate the analyzed time windows, respectively. The violin plots depict the distribution of the data per condition for the electrodes and the time window chosen for analyses. The mean topography was calculated as *mean* (VR‐Old, PC‐Old) *minus* CR. *p ≤ 0.029, ***p < 0.001, *n.s.* = not significant after correcting for multiple comparisons.

The rmANOVA revealed no significant main effect of the factor *Condition* (*F*(1.61, 38.55) = 1.62, *p* = 0.214, *η* ^2^ = 0.06; *BF*_10_ = 0.38) on the FN400. Descriptively, both kinds of engrams yielded a more negative response than correctly rejected items, deviating from previous, well‐established findings that the FN400‐response is less negative going to old stimuli (see Figure 3). However, the LPC response differed depending on *Condition* (*F*(1.48, 35.51) = 9.15, *p* = 0.002, *η* ^2^ = 0.28; *BF*_10_ = 64.71) and was thus compared between conditions using post hoc *t*‐tests. Both PC‐ and VR‐engrams yielded significantly more positive responses compared to correctly rejected items at parietal sensors, i.e., exhibited the conventional parietal old/new effect (PC‐Old vs. CR: *t*(24) = 1.99, *p* = 0.029, *d* = 0.40; *BF*_10_ = 1.13: VR‐Old vs. CR*: t* (24) = 6.68, *p* < 0.001, *d* = 1.34; *BF*_10_ > 9999; see Figure 3). However, no significant difference was found between VR‐engrams and PC‐engrams after correction for multiple comparisons (*t*(24) = −1.76, *p* = 0.045, *d* = 0.34; *BF*_10_ = 0.81).

#### 3.3.2 Source Identification Task

**FIGURE 4:**
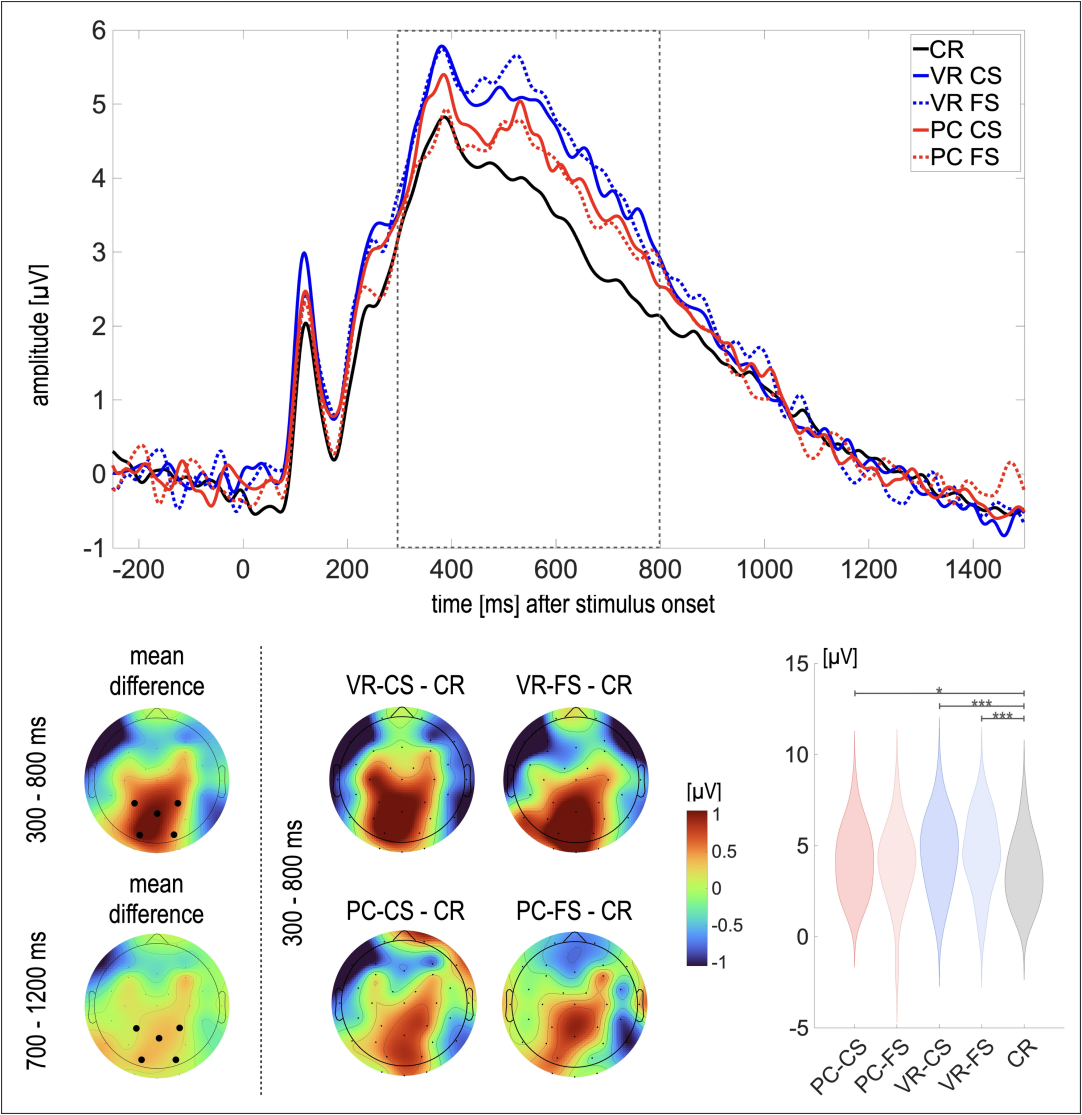
Amplitude distribution and line plots of the source memory effects. *Note.* The black dots in the topographies indicate the electrodes chosen for analyses (Pz, CP1, CP2, PO3, and PO4), and the grey dotted line indicates the time window analyzed using post hoc *t*‐tests. No post hoc tests were conducted for the 700–1200 ms time window, as the rmANOVA did not reach significance. The violin plots depict the distribution of the data per condition for the electrodes chosen for analyses in the time window from 300 to 800 ms. The amplitude distribution of the mean difference was calculated as *mean* (VR‐CS, VR‐FS, PC‐CS, and PC‐FS) *minus CR* per time window. VR‐CS = VR‐encoded item with correct source identification, VR‐FS = VR‐encoded item with false source identification, PC‐CS = PC‐encoded item with correct source identification, PC‐FS = PC‐encoded item with false source identification, CR = Correct rejection. *p ≤ 0.019, ***p < 0.001.

The 2 × 5 rmANOVA indicated a significant main effect of *Time Window* (*F*(2, 24) = 121.02, *p* < 0.001, *η* ^2^ = 0.84; *BF*_10_ > 9999), and a significant interaction of *Time Window* and *Condition* (*F*(2.44, 58.51) = 3.88, *p* < 0.019, *η* ^2^ = 0.14; *BF*_10_ > 9999) on the LPN, while no main effect of *Condition* (*F*(1.90, 45.67) = 2.47, *p* = 0.098, *η* ^2^ = 0.09; *BF*_10_ = 1.42) was found. The interaction was followed up by a 1 × 5 rmANOVA per time window, including only the factor *Condition*. Surprisingly, no significant effect of *Condition* was found for the later, conventional LPN time window (*F*(1.93, 46.25) = 0.82, *p* = 0.444, *η* ^2^ = 0.03; *BF*_10_ = 0.09) but only for the early time window, rather linked to the FN400 and LPC effects (*F*(2.07, 49.74) = 3.50, *p* = 0.036, *η* ^2^ = 0.13; *BF*_10_ = 3.62). Consequently, comparisons of the individual old conditions (VR‐CS, VR‐FS, PC‐FS, and PC‐CS) to CR were carried out for the early time window. PC‐CS, VR‐CS, and VR‐FS exhibited a significantly more positive response compared to CR (all *t*s > 2.5, all *p*s < 0.009, *d*s = [0.49; 1.0]; PC‐CS vs. CR: *BF*_10_ = 2.82, VR‐CS vs. CR: *BF*_10_ = 860.82, VR‐FS vs. CR: *BF*_10_ = 37.93; see Figure 4). However, the response to PC‐FS did not significantly differ from the response to CR (*t*(24) = 1.07, *p* = 0.15, *d* = 0.21; *BF*_10_ = 0.35).

## 4 Discussion

The study at hand aimed to unravel potential context‐transfer effects on the relative contribution of the processes underlying episodic memory, i.e., familiarity and recollection, to retrieval depending on the encoding condition. To this end, we replicated the design of a preceding study from our working unit (Kisker, Soethe, et al. 2025), which was based on a source memory paradigm. Participants incidentally encoded everyday objects in VR and on a PC. In contrast to the preceding study, the unannounced recognition task was carried out in VR instead of on a desktop to test item and source memory. We hypothesized that if the results of the preceding study were reversed in a 1:1 manner, i.e., if PC‐engrams with incorrect source attribution were associated with attenuated familiarity compared to VR‐engrams with incorrect source attribution, these shifts in recognition processes would be primarily based on context‐transfer effects. However, the results of the present study go well beyond mirroring previous results: Whereas the electrophysiological correlates of recognition memory indeed indicated attenuated familiarity of PC‐engrams, the same held true for VR‐engrams with respect to the FN400. In contrast, both conditions were linked to a substantial effect in the LPC related to recollection, whereas the correlate of the retrieval of contextual details related to source memory effects, i.e., the LPN, was not observed for either condition.

Contrary to our hypotheses, memory performance did not differ depending on encoding modality. Despite a trend towards increased memory performance for VR‐engrams, these differences did not reach significance after correcting for multiple testing. These results cannot be traced back to differences in encoding duration or success, as no differences between the encoding modalities were observed. Based on the findings of Johnsdorf et al. (2025) that the transfer from 2D to 3D results in reduced memory performance compared with matched encoding and retrieval modalities, it was expected that PC‐engrams would be more challenging to retrieve under VR conditions. With respect to the marginal trend towards better performance regarding VR‐engrams, our results neither strongly contradict nor strongly support the assumption that 2D‐3D transfer is less successful than 3D‐2D transfer. However, in our study, isolated everyday objects had to be recalled rather than entire scenes (Johnsdorf et al. 2025). This suggests that the potentially enhanced memorability of VR‐engrams, or the mitigated transfer of 2D to 3D scenes, may be based on an interaction between the encoding modality and stimulus complexity. Moreover, these results suggest that VR‐engrams might not necessarily be retrieved more successfully but may instead compensate for adverse contextual effects.

Beyond behavioral performance, the results are consistent with the preceding study (Kisker, Soethe, et al. 2025) in that the same theoretical conclusions about memory performance and the underlying processes would be derived from electrophysiological correlates tied to item memory, independent of the encoding modality. Similar contributions of recollection for both kinds of engrams were mirrored in the LPC. A stronger effect size was observed for VR‐engrams. However, their comparison to PC‐engrams did not reach significance after correcting for multiple testing, although indicating a trend towards more substantial recollection‐related effects in the VR condition. Surprisingly, no significant old/new effect was observed in the FN400, primarily reflecting item familiarity, irrespective of the encoding modality. This particular pattern of results might be explained by attenuated familiarity, increased recollection masking mere familiarity, or both.

In particular, the preceding study (Kisker, Soethe, et al. 2025) discussed whether VR‐engrams miss some information from encoding that is available for PC‐engrams during retrieval on a desktop. With respect to the encoding‐specificity principle, which states that memory retrieval is facilitated when the cues present during encoding and retrieval are matched (Tulving and Thomson 1973), there is less cue overlap when retrieving VR‐engrams on a 2D monitor. This context mismatch sufficiently explained why familiarity was attenuated for VR‐engrams with false source attribution (Kisker, Soethe, et al. 2025). In contrast, the present results indicated attenuated reliance on familiarity for both VR‐ and PC‐engrams. Given potential dependence on encoding specificity, the VR‐based retrieval condition might not result in a mismatch at the level of dimensionality but rather in a visual context mismatch across both encoding conditions, leading to decreased reliance on familiarity for both kinds of engrams. However, this explanation is challenged by the design of the preceding study, in which the retrieval condition (stimuli against a grey background) was visually distinct from the encoding conditions (stimuli on a table), yet elicited a canonical FN400 old/new effect across conditions. Consequently, the visual design of the retrieval context is unlikely to have served as a decisive retrieval cue in either encoding condition across studies.

Beyond the visual content, context‐related effects might be sensitive to hardware features like refresh rate and display resolution. For example, most VR HMDs used in memory studies have higher refresh rates than conventional desktops (e.g., 90 Hz vs. 60 Hz) but do not indicate that refresh rate alone significantly impacts memory performance, except for cases in which low refresh rates disrupt perception or cause discomfort (Cho et al. 2025; Denes et al. 2020). Similarly, there are no clear indications that higher display resolution improves memory performance if visual clarity of the presentation is given (Chocholáčková et al. 2023). Albeit larger display sizes generally increase users' sense of immersion, there is little difference in immersion between medium and large screens (Rigby et al. 2016). Whereas different manipulations of immersion might generally affect memory performance (Smith and Mulligan 2021), the use of a relatively large screen for PC‐based encoding in our study was intended to control for comparable stimulus presentation across conditions, beyond differences in dimensionality. Overall, the hardware features of the encoding phase are unlikely to explain the observed effects during retrieval.

As a variant of the encoding‐specificity principle, the concept of transfer‐appropriate processing holds that, rather than a cue‐match between encoding and retrieval, a match between the processes engaged during both phases is crucial for enhancing memory performance (Morris et al. 1977). Although both explanations are not mutually exclusive, transfer‐appropriate processing might explain the results if VR‐based retrieval—in contrast to PC‐based retrieval—activates processes distinct from those engaged during the encoding of both VR‐ and PC‐engrams. Otherwise, it would be expected that VR‐engrams and VR‐based retrieval overlap more strongly than PC‐engrams and VR‐based retrieval, which is why the principle would not explain the attenuated electrophysiological correlate of familiarity for VR‐engrams under these circumstances.

An alternative explanation is that recollection was facilitated for both types of engrams, thereby masking the effects usually associated with item familiarity. For example, the LPC is more pronounced for engrams retrieved with high confidence, albeit different confidence levels would not necessarily predict different levels of the LPC (Addante et al. 2012, 2020; Woroch et al. 2019). However, current evidence indicates that accurate source judgments are supported by varying degrees of recollection, as indicated by variations of the LPC depending on the confidence or strength (Woroch et al. 2019) and correctness (Leynes and Phillips 2008) of source memory. With respect to these findings, participants in our study were most confident about CRs of new items and VR‐engrams retrieved with correct source attributions, followed by PC‐engrams independent of source attribution, and, lastly, VR‐engrams with incorrect source attributions. However, this pattern of confidence was not reflected in the LPC when tied to the correctness of the source attributions. In contrast, the explorative analyses of the time window from 300 to 800 ms descriptively yielded the strongest response to both VR‐engrams, followed by PC‐engrams, and thus stand in contrast to findings indicating graded responses of the LPC depending on source memory. Interestingly, the LPC did not differ between PC‐engrams with incorrect source attribution and correctly rejected items. This result is consistent with the hypothesis that if dependent on context effects, the respective effects of the previous study would be reversed in the present study. In other words, while VR‐engrams with incorrect source attribution stood out when retrieved on a desktop (Kisker, Soethe, et al. 2025), PC‐engrams with incorrect source attribution stand out when retrieved in VR, underscoring the potential role of a context mismatch in this effect.

However, the finding of attenuated familiarity of VR‐engrams was based on the LPN in the previous study. In contrast, PC‐engrams with incorrect source attribution stand out regarding the LPC in the present study. Moreover, the characteristics of LPN were not observed as expected: Following the LPC, the amplitude levels returned approximately to the pre‐stimulus baseline level. Neither the observed topographical distribution nor the amplitude levels were characteristic of the LPN in the subsequent time window. Instead, a differentiation depending on the source attribution was observed during the time window of the FN400 and LPC old/new effects at parietal sensors. Omitting timing considerations, this result represents the opposite pattern observed in the previous study. However, the previous study attributed the results to the search for and reactivation of contextual details based on the LPN (Johansson and Mecklinger 2003; Mecklinger et al. 2007, 2016). In contrast, the observed differentiation in the LPC in the current study more directly indexes recollection. Thus, the two patterns of results are not interchangeable, although both establish links to contextual information.

Recent updates introduce a subdivision of familiarity into item familiarity and context familiarity, respectively (Addante et al. 2024). This dissociation is supported by a broad central negativity (BCN) associated with items recognized with low confidence but correct source attribution. This effect is dissociable from the LPC and FN400 (Addante et al. 2024) as well as from the LPN (Addante et al. 2024; Mecklinger et al. 2016), underscoring its distinction from item familiarity and recollection. However, similar to the preceding study (Kisker, Soethe, et al. 2025), the present data did not match the characteristics of the BCN as determined by visual inspection as a countercheck. An unintended measurement of the BCN thus cannot explain the present results.

Overall, and in contrast to previous results, the study at hand indicates attenuated contributions of item familiarity and, respectively, strong contributions of recollection to retrieval, independent of encoding modality. Ultimately, only the retrieval condition was varied between studies, whereas the encoding phase was kept strictly identical. The discrepancies in the results, therefore, go beyond mere context‐transfer effects between encoding and retrieval, rendering the retrieval condition a decisive factor. For example, retrieval in VR might increase the reliance on recollection—including but going beyond familiarity—by offering detailed and sensory‐rich retrieval cues (e.g., Kisker, Johnsdorf, et al. 2025a; Parsons 2015; Serino and Repetto 2018; Wilson and Soranzo 2015). These, in consequence, might facilitate reconstructive processes of either engram during memory retrieval, which would in turn be consistent with the assumption that the efficiency of cognitive processing is increased under VR conditions, thereby consuming fewer cognitive resources (Dan and Reiner 2017; Johnsdorf et al. 2023; Kisker, Johnsdorf, et al. 2025b; see also Kisker et al. 2021b).

However, as discussed above, most studies focus on encoding‐specificity rather than on the effects of the retrieval cue's modality by means of dimensionality. They would explain increased recollection for VR‐engrams or attenuated familiarity of PC‐engrams when retrieved in VR. Although there are indications that familiarity and recollection are affected by the match between encoding and retrieval, the evidence is mixed (see, e.g., Curran and Dien 2003; Ecker et al. 2009; Nyhus and Curran 2009). For example, sensitivity to study‐test match depending on the task's affordances and the materials has been reported for either process (Ecker et al. 2009; Yonelinas 2002), whereas some studies find both familiarity and recollection to be robust when switching between, e.g., auditory and visual presentation between encoding and retrieval (Curran and Dien 2003; Yonelinas 2002). The specific effects of varying the retrieval cue by switching between 2D and 3D presentations of the same stimuli have not been systematically studied to the best of our knowledge. Therefore, it remains to be explored whether VR‐based retrieval ultimately attenuates familiarity and predominantly engages recollection independent of encoding conditions.

## 5 Limitations and Future Directions

In the previous study (Kisker, Soethe, et al. 2025), retrieval was performed on a desktop for both types of engrams to ensure similar sensory input across conditions, allowing for attributing the observed results to the experimental manipulations during encoding. Following the same rationale, retrieval of both types of engrams was performed in VR in the present study. Beyond the intended identification of potential context‐transfer effects, the results indicate that the retrieval condition per se may affect the relative contributions of recollection and familiarity to retrieval. To isolate potential influences of the retrieval condition, a future study should carry out a full cross‐comparison of the encoding and retrieval conditions (for a similar procedure, see Johnsdorf et al. 2025).

Moreover, the electrodes and time windows for the analysis of the LPN were derived from preceding literature and intended to replicate the analyses carried out by Kisker et al. (2025) for a precise comparilowing the same rationale, retrieval of both types of engrams was performed in VR in the present study. Beyond the intended identification of potential context‐transfer effects, the results indicate that the retrieval condition per se may affect the relative contributions of recollection and familiarity to retrieval. To isolate potential influences of the retrieval condition, a future study should carry out a full cross‐comparison of the encoding and retrieval conditions (for a similar procedure, see Johnsdorf et al. 2025).

Moreover, the electrodes and time windows for the analysis of the LPN were derived from preceding literature and intended to replicate the analyses carried out by Kisker et al. (2025) for a precise comparison. Notably, our data did not correspond to the characteristics of the LPN using these parameters, i.e., no negative deflection, maximal at posterior sensors, with an onset around 800 ms was observed. While further studies investigate a later time window, i.e., 1200–1800 ms, which is thought to reflect the maintenance of the retrieved episode (e.g., Herron 2007; Mecklinger 2000; Mecklinger et al. 2007), this time window was not adequate for our analyses as it is interrupted by the offset of the stimulus and the participants' ratings. However, it cannot be ruled out that the LPN would have differentiated at a later stage depending on the source attributions, although the visual inspection of the data did not indicate this until 1500 ms after stimulus onset.

## 6 Conclusions

In summary, the present results indicated similar contributions of the processes underlying episodic memory, i.e., familiarity and recollection, to retrieval, independent of the encoding condition, when retrieved under VR conditions, as reflected in the FN400 and LPC. While effects on engrams retrieved without their correct source might to some degree depend on context‐transfer effects, familiarity was attenuated across encoding conditions, thus going beyond effects of encoding specificity. The present findings, building upon previous work (Kisker, Soethe, et al. 2025), demonstrate that disparities between VR‐ and PC‐engrams depend on the specific combination of encoding and retrieval modalities.

## Author Contributions

**Joanna Kisker:** conceptualization, formal analysis, investigation, methodology, project administration, software, visualization, writing – original draft, writing – review and editing. **Marius Soethe:** investigation, software, writing – review and editing. **Merle Sagehorn:** writing – review and editing. **Thomas Gruber:** conceptualization, formal analysis, methodology, resources, supervision, writing – review and editing.

## Funding

The authors have nothing to report.

## Ethics Statement

The studies involving human participants were reviewed and approved by the local ethics committee of Osnabrück University, Germany (reference: Ethik‐36/2024). The participants provided their written informed consent to participate in this study.

## Conflicts of Interest

The authors declare no conflicts of interest.

## Supporting information


**Table S1:** Trials per condition.
**Table S2:** Descriptive and inferential statistics regarding the paired comparisons of the behavioral data of the encoding phase.
**Table S3:** Descriptive statistics regarding the paired comparisons of the behavioral and electrophysiological data of the retrieval phase.

## Data Availability

The datasets presented in this study are available in the online repository OSF. The file containing the data was updated once (November 26, 2025) during the review process: https://osf.io/4sc9f/overview?view_only=5d947819d535483c93877bc8a5be7aff.
