# Supplementary material for: Beyond Context‐Transfer Effects: Attenuated Familiarity During Virtual Reality‐Based Retrieval Across Different Encoding Modalities
Source: Eur J Neurosci. 2026 Mar 6;63(5):e70447. doi: 10.1111/ejn.70447 (PMC12964179; doi:10.1111/ejn.70447)
Supplement: Supplementary file 1 — Table S1: Trials per condition. Table S2: Descriptive and inferential statistics regarding the paired comparisons of the behavioral data of the encoding phase. Table S3: Descriptive statistics regarding the paired comparisons of the behavioral and electrophysiological data of the retrieval phase. [file EJN-63-0-s001.docx]

Supplementary Table 1. Trials per condition.

| *task* | *condition* | *average* | *min* | *max* |
| --- | --- | --- | --- | --- |
| Old/New | VR | 66.32 | 46 | 80 |
|  | PC | 64.52 | 46 | 81 |
|  | CR | 74.56 | 54 | 90 |
| Source identification | VR-CS | 40.52 | 12 | 58 |
|  | VR-FS | 25.80 | 6 | 35 |
|  | PC-CS | 38.16 | 19 | 63 |
|  | PC-FS | 26.36 | 13 | 47 |
|  | CR | 74.56 | 54 | 90 |

Supplementary Table 2. Descriptive and inferential statistics regarding the paired comparisons of the behavioral data of the encoding phase.

| *comparison* |  | Paired Differences | | | | |
| --- | --- | --- | --- | --- | --- | --- |
|  |  | Mean | Std. Deviation | Std. Error Mean | 95% Confidence Interval of the Difference | |
|  |  |  |  |  | Lower | Upper |
| Response time | correct answers vs. false answers | -255.330 | 247.169 | 46.710 | -351.167 | -159.480 |
| Correct answers | VR vs. PC | -2.571 | 9.788 | 1.850 | -6.367 | 1.220 |

Supplementary Table 2. Descriptive statistics regarding the paired comparisons of the behavioral and electrophysiological data of the retrieval phase.

| *comparison* | | Paired Differences | | | | |
| --- | --- | --- | --- | --- | --- | --- |
|  |  | Mean | Std. Deviation | Std. Error Mean | 95% Confidence Interval of the Difference | |
|  |  |  |  |  | Lower | Upper |
| d-Prime | VR vs. PC | .028 | .081 | .015 | -.004 | .059 |
| Correct source identifications | VR vs. PC | .013 | .199 | .038 | -.064 | .090 |
| Confidence | CR vs. VR-CS | .038 | .314 | .059 | -.084 | .159 |
|  | CR vs. VR-FS | .438 | .303 | .057 | .320 | .555 |
|  | CR vs. PC-CS | .271 | .343 | .065 | .138 | .404 |
|  | CR vs. PC-FS | .270 | .323 | .061 | .145 | .395 |
|  | VR-CS vs. VR-FS | .400 | .314 | .059 | .278 | .522 |
|  | PC-CS vs. PC-FS | -.001 | .403 | .076 | -.157 | .155 |
|  | VR-CS vs. PC-CS | .233 | .393 | .074 | .081 | .386 |
|  | VR-FS vs. FC-CS | -.168 | .313 | .059 | -.290 | -.047 |
| LPC | VR vs. CR | 1.45 | 1.087 | .218 | 1.004 | 1.902 |
|  | PC vs. CR | .782 | 1.968 | .394 | -.031 | 1.594 |
|  | VR vs. PC | -.671 | 1.901 | .380 | -1.455 | .114 |
| LPN-electrodes, 300 - 800ms | PC-CS vs. CR | .623 | 1.244 | .249 | .113 | 1.140 |
|  | PC-FS vs. CR | .464 | 2.169 | .434 | -.431 | 1.360 |
|  | VR-CS vs. CR | 1.014 | .982 | .196 | .608 | 1.419 |
|  | VR-FS vs. CR | 1.113 | 1.469 | .294 | .506 | 1.719 |
